# Retained Curved Needle After Balloon Kyphoplasty: A Complication with a Novel Device and Its Management

**DOI:** 10.7759/cureus.4367

**Published:** 2019-04-02

**Authors:** Neal A Shah, Eric Catlin, Navdeep Jassal, Osama Hafez, Devang Padalia

**Affiliations:** 1 Anesthesia and Interventional Pain Management, H. Lee Moffitt Cancer Center and Research Institute, Tampa, USA; 2 Physical Medicine and Rehabilitation, University of South Florida, Tampa, USA; 3 Pain Management, University of South Florida, Tampa, USA; 4 Anesthesiology, H. Lee Moffitt Cancer Center and Research Institute, Tampa, USA; 5 Anesthesia and Interventional Pain Management, H. Lee Moffitt Cancer Center and Research Institute, Ormond Beach, USA

**Keywords:** vertebral augmentation, vertebral compression fracture, complication, kyphoplasty, foreign body, lumbar pain

## Abstract

To date, no case studies specifically describing a curved kyphoplasty needle becoming lodged in the vertebral body with the inability to be withdrawn have been reported. We describe a case involving a single level balloon kyphoplasty with a curved coaxial needle during which the cement delivery device could not be removed after cavity filling. In this case, a board-certified interventional pain management specialist was performing balloon kyphoplasty for an L2 osteoporotic vertebral compression fracture. The tools utilized in this procedure included flexible curved instruments designed to traverse the vertebral body and achieve uniform cement distribution through a unipedicular approach. Cannulation and cavity formation were completed without issue. Upon conclusion of cement filling, the curved cement delivery device was unable to be removed. After several attempts to remove the needle and consultation with both the device company and local spine surgeons, it was agreed that the device should be cut at the level of entry into the pedicle and left as a retained foreign object. The involved area was surgically exposed, the retained instrument was cut flush to the pedicle, and the free portion was removed without further complication. The patient followed up in clinic several months later without evidence of neurologic complications. Considerations when using a curved kyphoplasty device and a method of resolution when faced with the inability to remove such an instrument are discussed.

## Introduction

Osteoporotic vertebral compression fractures (OVCF) are a source of significant morbidity. The incidence in the United States has been estimated to be greater than 500,000 cases annually, with a projected increase of greater than 50% by 2025 [1]. OVCF has been shown to have a substantial impact on patients’ quality of life after injury, and this tremendous burden has led to the pursuit of innovative treatment options [2].

In the 1980s, percutaneous vertebroplasty (PVP) was introduced for the treatment of aggressive hemangiomas [3]. Implementing this technique and technology for OVCF soon followed. Percutaneous kyphoplasty (PKP), a variation of PVP, was developed several years later with the premise of utilizing an inflatable balloon tamp to create a cavity. This allowed for the augmentation of the vertebral body height while permitting a lower pressure for cement injection [4]. This low-pressure technique has allowed for higher viscosity cement to be utilized. Although cement leakage has remained one of the most common complications with these methods, the rates of cement leakage with PKP have been reported to average 18.4% as compared with 59.7% in PVP [5]. Clinicians initially utilized a bilateral transpedicular approach with these procedures, however, a unipedicular approach has recently been developed and has gained increasing acceptance. The unilateral approach provides the advantage of reduced operative and anesthesia time, as well as a reduction in the costs of balloon tamps, cannulas, and needles. Nevertheless, recent reviews have identified no significant difference in clinical outcomes when comparing the unilateral and bilateral approaches [6-7].

Vertebral augmentation procedures continue to evolve with new generations of cement formulae being designed to provide better working properties, structural integrity, and bioactive variations to induce new bone formation [8]. New cavity-creation techniques also continue to expand. Curette-like tips, articulating osteotomes, and curved coaxial needles with flexible balloon systems provide additional options to better tailor treatment to individual patients [8-9]. Unfortunately, as new techniques are developed, they are often accompanied by new complications. We present a case of L2 kyphoplasty performed with a curved coaxial needle and balloon system during which the curved needle became lodged in the vertebral body and was unable to be removed. The needle was subsequently cut off at the level of the pedicle and left as a retained foreign body.

## Case presentation

**Figure 1:**
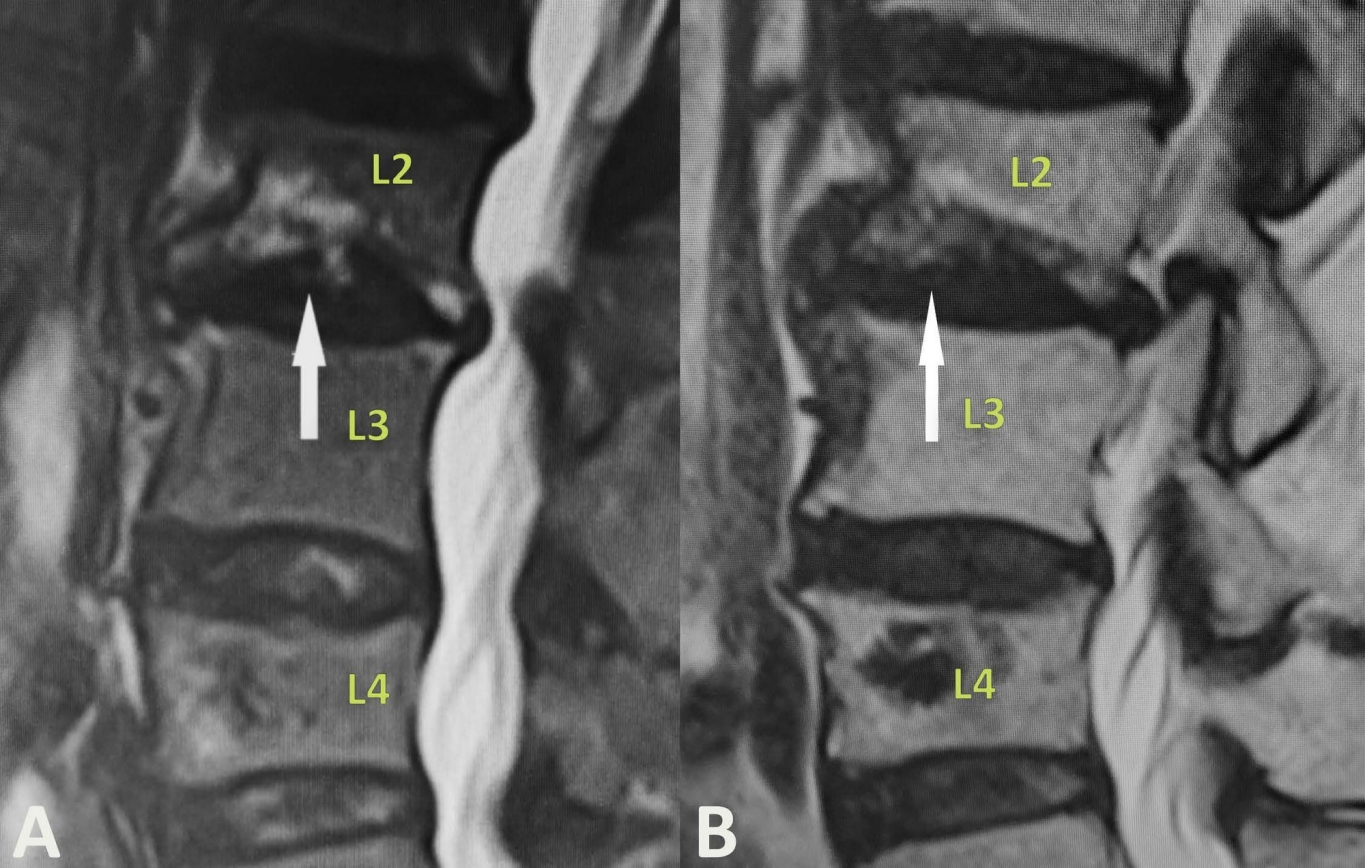
Sagittal short-TI inversion recovery (A) and standard T2-weighted (B) MRI scans show an acute L2 compression fracture with inferior endplate involvement

An 82-year-old woman with known osteoporosis presented with several weeks of unrelenting axial lower back pain. After conservative management with pain medication and rest, she was referred to a pain management clinic for further evaluation. On examination, the patient had tenderness to percussion at the 2nd lumbar vertebral body (L2) without evidence of radiculopathy. T2-weighted magnetic resonance imaging (MRI) revealed an acute compression fracture with inferior endplate involvement at the level of L2 (Figures 1A-1B). After extensive discussion, the patient elected to proceed with L2 balloon kyphoplasty.

The patient was brought to the operating room, placed in the prone position, and prepped and draped in the standard fashion. Under direct fluoroscopic guidance, the left L2 pedicle was successfully cannulated on the first attempt using a 10-gauge access cannula. The trocar-tip stylet was removed and a curved coaxial needle with a radiopaque introducer was inserted through the access cannula and advanced to the proper midline position within the L2 vertebral body (targeted site location). The curved coaxial needle was then removed, leaving the introducer and access cannula in place. A flexible kyphoplasty balloon was then placed through the introducer and access cannula. Cavity creation was performed with the inflation of the balloon system and completed without complications. The balloon was deflated and removed simultaneously with the introducer, leaving just the access cannula in place. The curved coaxial needle was primed with polymethylmethacrylate (PMMA) and reinserted into the vertebral body through the access cannula. A total of 2.5 cc of PMMA was used and an even fill was seen on fluoroscopic imaging.

Attempts were made to remove the curved coaxial needle from the access cannula, but it was found to be fastened in place. The time elapsed since cement creation was nine minutes. Multiple attempts were made to remove the needle but it remained firmly in place despite the use of a mallet to loosen the instrument. After consultation with the physician representatives of the device company and two independent spine surgeons, the decision was made to dissect down to the level of the pedicle and cut the flexible needle at the level of entry into the bone.

**Figure 2:**
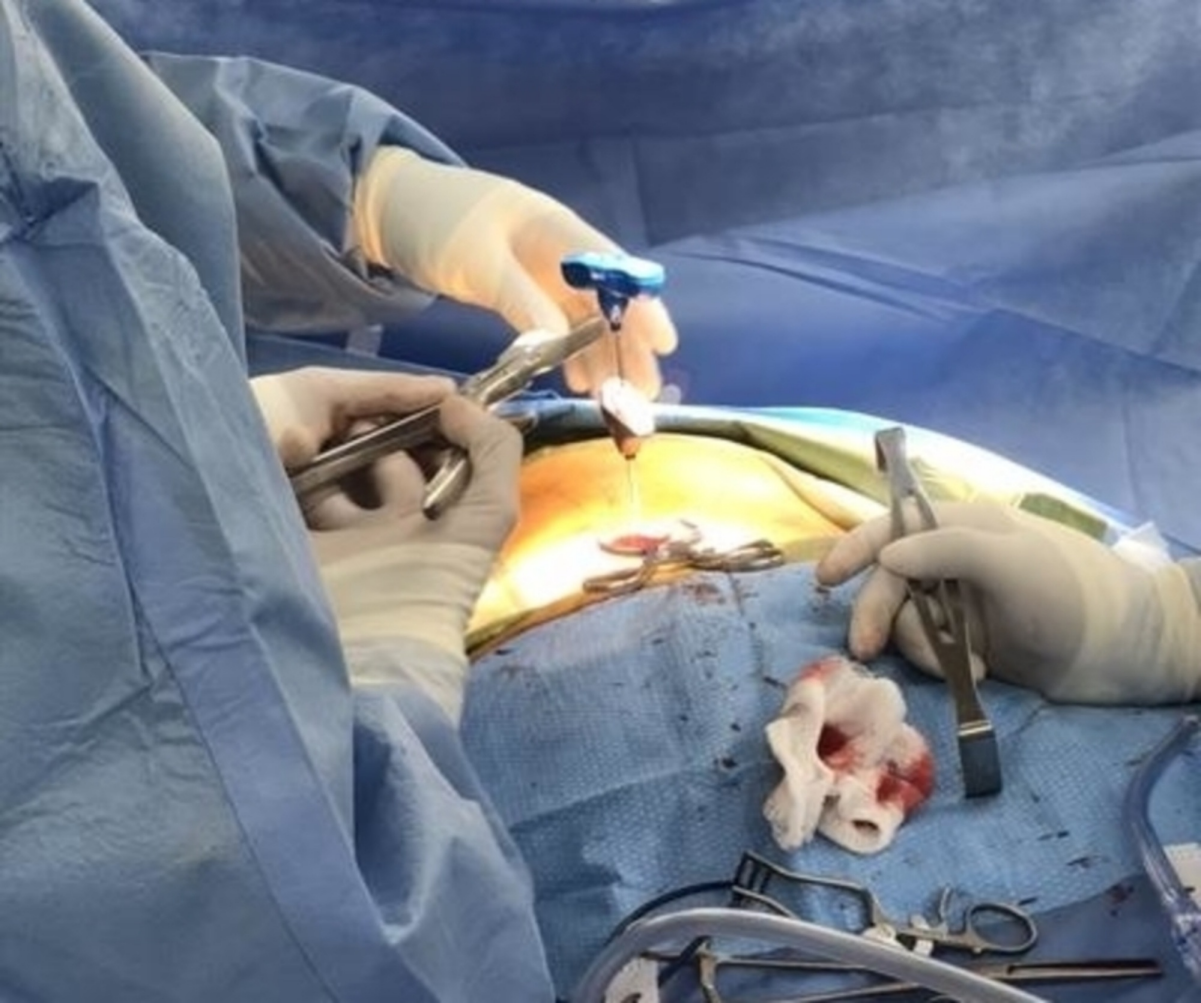
Cutting the handle of the stuck curved coaxial needle to allow the removal of the surrounding access cannula

**Figure 3:**
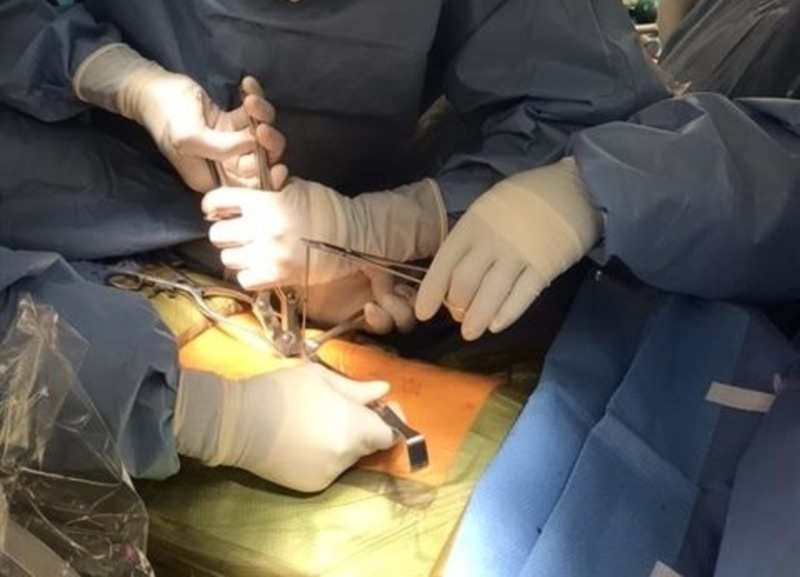
Cutting the stuck needle at the level of entry into the pedicle after the access cannula has been removed

**Figure 4:**
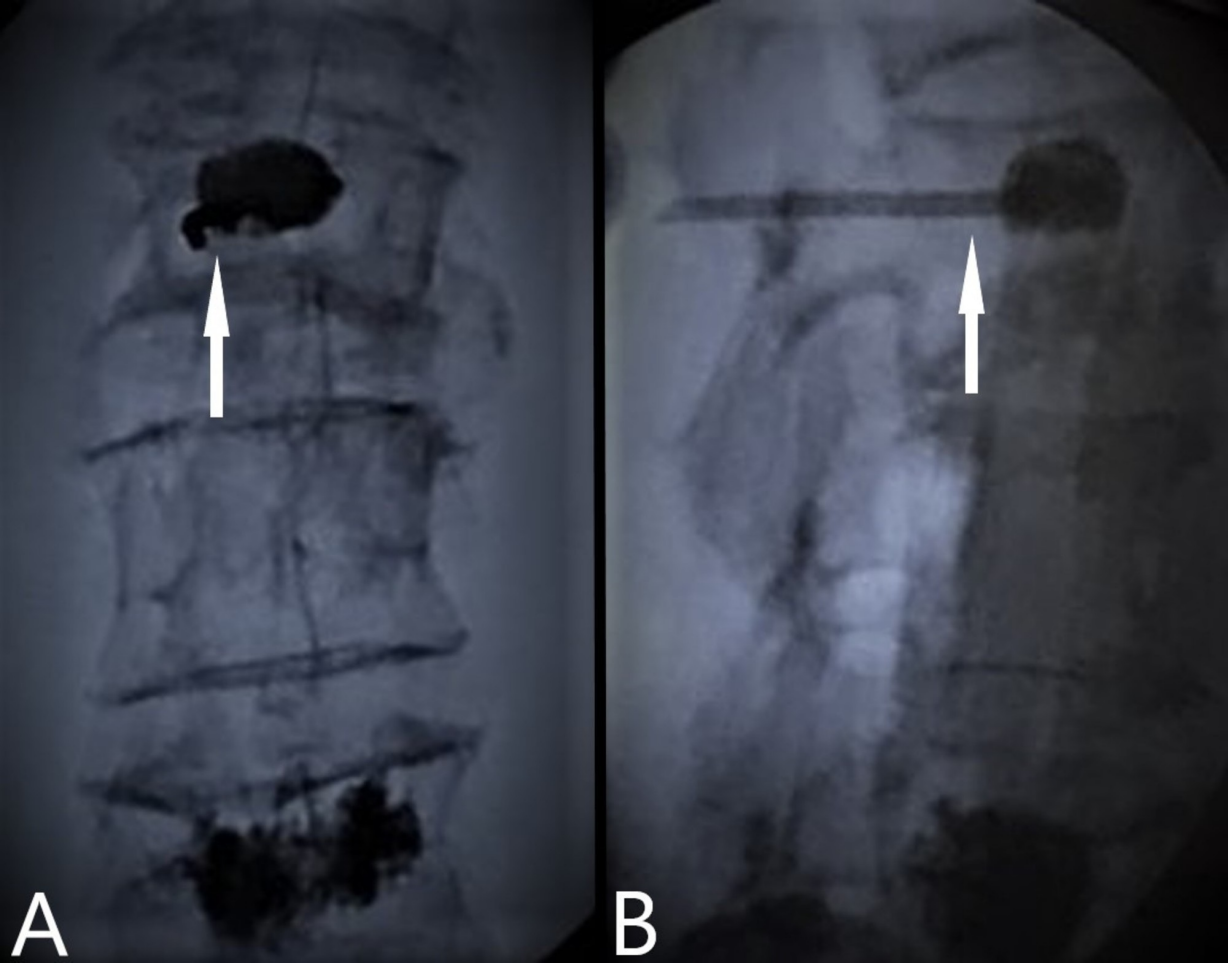
Anteroposterior (A) and lateral (B) views of retained device

After additional local anesthetic was injected, the subcutaneous layer was dissected and muscle tissue was retracted. The proximal handle of the curved coaxial needle was cut with a bone rongeur (Figure 2) and the access cannula was removed from around it. The curved needle was cut to be as flush as possible to the level of the left L2 pedicle (Figure 3) and palpated for sharp edges or protrusions from the pedicle border. Profuse irrigation was completed with bacitracin and the pocket and incision were closed in layers and dressed. Final images were then taken (Figures 4A-4B).

The patient tolerated the procedure well and had no postoperative neurological compromise or injury. Her recovery period was uneventful. At all follow-up appointments through a 12-month period, the patient had sustained relief from her back pain and remained without evidence of neurological complications.

## Discussion

Percutaneous vertebral augmentation techniques continue to develop with time. In addition, innovative tools and materials continue to emerge, addressing various fracture types, cavity-creation methods, and cement compositions. The objective of all these techniques is to reduce complications while allowing for the creation of a stable and reinforced vertebral body, ultimately reducing the patient’s pain and increasing functional activity. This case provides an example of an intraoperative complication occurring during the use of a novel instrument added to a percutaneous balloon kyphoplasty kit. The use of a curved coaxial needle in combination with the curved balloon system allows for a unipedicular approach while theoretically generating an even distribution of cement without requiring bipedicular cannulation. Although curved or articulating tools provide greater flexibility for traversing the vertebral body, in this case, the curved shape of the tool may have contributed to the inability to remove the instrument from the vertebral body as easily as a straight device. It is also possible that, despite being within the accepted PMMA working time when device retraction was attempted, the cement hardened around the needle tip and led to this complication. It was determined that further attempts at removal would potentially result in damage to the vertebral body and pedicle, therefore, the portion of the device in the vertebral body was left in place.

The incidence of retained foreign objects (RFO) after surgery has been estimated to occur at a rate of approximately one per 5500 operations. The vast majority of reported RFOs are sponges, and most are atraumatically removed after being identified on routine postoperative imaging. Very few RFOs are not removed after discovery, specifically in cases where removal would pose a greater risk to the patient than allowing the RFO to remain in place [10]. Retrieval of broken spinal hardware can be a particularly difficult process, and there is no single best approach to reacquiring this equipment [11]. While there are reports of retrieving broken Jamshidi needle fragments [12] and a fractured pedicle cannulation probe [13] in the literature, to our knowledge, there is only one other case reported that discusses the approach to managing irretrievable percutaneous vertebral augmentation hardware. In this particular case, a trocar was stuck in the vertebral body with a solidified cement mass attached to the distal tip, resulting in an inability to remove the device. A similar procedure was utilized to address the complication, resulting in a similar RFO [14]. While RFOs that are in contact with neural structures can become symptomatic, those that are confined without breach of bony cortical walls can theoretically be safely left in place due to the low likelihood of migration or cause of additional symptoms.

While leaving RFOs is never ideal, in this case, the likelihood of causing greater damage through forced removal was thought to be a higher risk than leaving the cemented object in place. The device that was left in place is made of similar material as standard spinal hardware implants. Some potential problems with leaving a metallic device in the vertebral body include increased difficulty in spine fixation surgeries and the potential for causing some distortion on future magnetic resonance imaging. In all follow-ups spanning over one year, the patient had not displayed any new or worsening symptoms since the procedure and has not required any repeat imaging.

## Conclusions

Careful consideration must be given to possible complications when utilizing new techniques for percutaneous vertebral augmentation. Although curved needles and instruments may facilitate enhanced cement coverage of a vertebral body, there is always the risk of increased difficulty in removal. Cutting a retained instrument at the level of the pedicle insertion site may be considered if the object is unable to be retrieved. This may be the safest course of action because the instrument remains encased within the vertebral body and, therefore, has a low chance of migration. This also precludes the need to attempt intrapedicular hardware removal, which could be associated with greater complications and damage to vital neighboring structures.
